# Distribution, host preference and infection rates of malaria vectors in Mauritania

**DOI:** 10.1186/1756-3305-2-61

**Published:** 2009-12-04

**Authors:** Ibrahima Dia, Hampate Ba, Sid Ahmed Ould Mohamed, Diawo Diallo, Baidy Lo, Mawlouth Diallo

**Affiliations:** 1 Unité d'Entomologie Médicale, Institut Pasteur de Dakar, BP 220, Dakar, Senegal; 2 Institut National de Recherches en Santé Publique, Nouakchott, Mauritania

## Abstract

This study reports for the first time on the distribution, host preference and infection rates of malaria vectors in Mauritania. It was conducted during an outbreak of Rift valley fever. Three anopheline species were reported. *An. arabiensis *was the predominant species observed in all regions whereas *An. pharoensis *and *An. funestus *were observed along the south border in the Senegal River valley where extensive irrigation schemes are present. The distribution limits of anopheline species were observed from the Senegal River basin in the Trarza region up to the south limit of the Saharan desert in Tidjikja city. Overall, all *An. funestus *and *An. pharoensis *were fed respectively on human and ovine hosts whereas the mean anthropophilic rate of *An. gambiae *s.l. was 53%. A low *Plasmodium falciparum *infection rate was observed for species of the *An. gambiae *complex (0.17%) represented mainly by *An. arabiensis*. Because of the specific nature of this investigation, longitudinal studies are essential to better characterize the malaria vectors and their respective role in malaria transmission.

## Findings

In Mauritania, malaria is a major public health concern in southern and south-eastern regions. In fact, it is clearly on the increase and significantly contributes to the increase in illness and mortality, especially in children under 5 years of age and pregnant women [1]. In spite of this importance, little is known about its vectors. The only published entomological studies date back 40 years [2,3], although limited entomological investigations were conducted during an outbreak of Rift Valley fever in several localities [4].

*An. gambiae *s.l. and *An. funestus *appear to be the dominant vectors of malaria. Their limits of distribution throughout the country are however unknown. This situation makes impossible the determination of the origin of malaria suspected cases in areas where malaria vectors are thought to be absent. One of the principal explanations for such suspected cases is that they have been imported. However, this hypothesis seems to be speculative with regards to some autochthonous cases observed [1]. The hypothesis that there is malaria transmission by anopheline species other than those already described above cannot be excluded. In many health centres, no parasitological analyses are performed and only clinical diagnoses are made. However, with the increasing number of cases attributed to malaria and the recurrent emergence of hemorrhagic fevers, the epidemiology of malaria is still unclear. For this reason we paid particular attention to the malaria infection rates of anthropophilic species during an entomological investigation of a recent outbreak of Rift valley fever [5].

**Figure 1:**
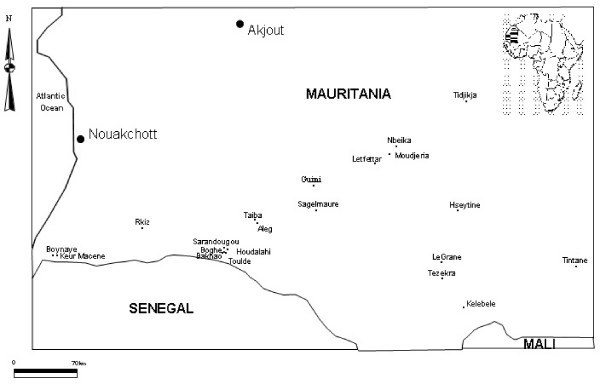
**Localisation of the study sites**.

Our survey covered 21 localities in 5 administrative regions during October and November 2003: Trarza (Boynaye, Keur Macene, Rkiz), Brakna (Aleg, Taiba, Guimi, Houdalahi, Bakhao, Boghe, Toulde, Sarandougou, Sagelmaure), Assaba (Kelebele, Tezekra, LeGrane, Hseytine) Tagant (Letfettar, Nbeika, Moudjeria, Tidjikja) and Hodh El Garbi (Tintane) (Figure 1). Mosquitoes were sampled by indoor pyrethrum spray catch method in selected dwellings in each locality. After collection, mosquitoes were sorted and identified using morphological keys. Blood meals from fed mosquitoes were blotted onto filter papers and the source was determined in the laboratory as described by Beier et al. [6]. All mosquitoes were stored individually in numbered vials with desiccant and crushed head-thoraces tested by ELISA for *Plasmodium falciparum*, *P. malaria *and *P. ovale *CS antigen [7]. The species from the *An. gambiae *complex and molecular forms of *An. gambiae *s.s. were identified by PCR according to Scott et al. [8] and Favia et al. [9] respectively.

In total, 647 anopheline specimens belonging to three species were collected. *An. gambiae *s.l. was the most common species (92%) followed by *An. pharoensis *(5%) and *An. funestus *(3%). *An. gambiae *was collected in all localities visited except in Moudjeria (Tagant region) where no mosquito was collected. All three species were collected in Trarza region, but *An. pharoensis *was observed only in Trarza and Brakna regions. The molecular identification of the species of the *An. gambiae *complex has revealed the predominance of *An. arabiensis*. *An. gambiae *M form was observed only in Assaba (1/6; 16.7%), Brakna (2/19; 10.5%) and Hodh El Garbi (2/6; 33.3%). The distribution limits of anopheline species ranged from the Senegal River Valley in Trarza region up to the south limit of the Saharan desert in Tidjikja city.

The predominance of *An. arabiensis *in the Mauritanian context is in agreement with the general distribution of this species in Africa [10]. *An. funestus *was observed only in Brakna region adjacent to the Senegal River basin where its comeback was recently reported [11], as it is also the case in the Sahelian region of Niger [12]. The development of irrigation schemes, following the implementation of the anti-salt dam at Diama (at the mouth of the Senegal River), has generated many breeding sites for *An. funestus *in this area. *An. pharoensis *whose vectorial role in malaria transmission was already suspected in the Senegal River delta [13] was found in both the Trarza and Brakna regions where large rice cultivation areas thought to be favourable for its aquatic stages are present.

The analysis of blood meals from blood fed females showed that no significant difference was observed between the anthropophilic rates of *An. gambiae *s.l. among the five regions (χ^2 ^= 4.57, df = 4, p = 0.34). All *An. funestus *and *An. pharoensis *had fed on human and ovine hosts respectively (Table 1).

**Table 1:** *Anopheles *species, resting densities, anthropophilic and circumsporozoite protein rates at different sites in Mauritania (October-November 2003).

Regions	*An. gambiae *s.l.				*An. funestus*				An. pharoensis			
			
	Collected	RD	AR	CSPR	Collected	RD	AR	CSPR	Collected	RD	AR	CSPR
Trarza	4	0.2	66.7 (3)	0 (4)	19	0.9	100 (3)	0 (19)	6	0.3	-	0 (6)
Brakna	142	5.1	37.8 (37)	0 (142)	-	-	-	-	28	1.1	0 (4)	0 (28)
Assaba	407	27	78.4 (37)	0.25 (394)	-	-	-	-	-	-	-	-
Hodh El Garbi	19	6.3	33.3 (3)	0 (19)	-	-	-	-	-	-	-	-
Tagant	22	2.2	88.9 (9)	0 (22)	-	-	-	-	-	-	-	-
		
All	594	8.2	60.7 (89)	0.17 (581)	19	0.9	100 (3)	0 (19)	34	0.7	0 (4)	0 (34)

RD = Resting Densities (mean number of females per room)

AR = Anthropophilic Rate (%)

CSPR = Circumsporozoite Protein Rate (%)

() = Number tested

Probably because of the low sample sizes, no *An. funestus *or *An. pharoensis *was found to be positive by ELISA for *P. falciparum*, *P. malariae *and *P. ovale *circumsopozoite antigen detection. However, of 394 females of the *An. gambiae *complex tested in Assaba, one *An. arabiensis *was positive for *P. falciparum*. The circumsporozoite rate was 0.25% for this region and 0.17% for the whole study area. The involvement of these species in malaria transmission in the Senegal River basin was recently observed [14]. This provides evidence for the possible involvement of this species in malaria transmission in this region, although the low infection rate contrasts with the suspected malaria burden in the region.

To our knowledge, this study reports, for the first time, on the distribution, host preference and infection rates of malaria vectors in Mauritania. With regard to these findings, and most notably the very low mosquito infection rates, it is clearly necessary to differentiate febrile cases attributed to malaria from other febrile infections, including the hemorrhagic fevers in the light of their recurrent emergence. However, the possibility that there is transmission of *P. falciparum *by other anopheline species such as *An*. *rhodesiensis *and *An*. *dthali *already reported in Mauritania [15] and transmission of other *Plasmodium *species (*P. vivax*) cannot be excluded. Moreover, because of the specific nature of this investigation, longitudinal entomological studies are essential in order to better characterize the malaria vector populations and to identify their respective roles in the transmission of human *Plasmodium*.

## Competing interests

The authors declare that they have no competing interests.

## Authors' contributions

HB, SAOM and DD contributed to sample collections and laboratory processing, BL participated to the study conception and coordination, ID and MD conceived, coordinated the study and drafted the manuscript. All authors read and approved the final manuscript.
